# A study of two generic health-related quality of life questionnaires—Nottingham Health Profile and Short-Form 36 Health Survey—and of coping in patients with sensory hyperreactivity

**DOI:** 10.1186/1477-7525-11-182

**Published:** 2013-10-29

**Authors:** Marja-Leena Kristofferzon, Ewa Ternesten-Hasséus

**Affiliations:** 1 Department of Health and Caring Sciences, Faculty of Health and Occupational Studies, University of Gävle, Gävle, Sweden; 2 Department of Public health and Caring Sciences, Uppsala University, Uppsala, Sweden; 3 Department of Respiratory Medicine and Allergology, Institution of Medicine, The Sahlgrenska Academy at University of Gothenburg, Gothenburg, Sweden

**Keywords:** Airway sensitivity, Chemical sensitivity, Coping, Environmental exposure, Gender issues, Health-related quality of life

## Abstract

**Background:**

Sensory hyperreactivity (SHR) is one explanation for airway symptoms induced by chemicals and scents. Little is known about health-related quality of life (HRQOL) and coping, in this group of patients. A study was done in patients with SHR to (1) compare the Nottingham Health Profile (NHP) and the Short-Form 36 Health Survey (SF-36) in regard to their suitability, validity, reliability, and acceptability; (2) evaluate how the patients cope with the illness; (3) assess whether there are differences between women and men with respect to HRQOL and coping; and (4) assess whether there are differences between patients and normative data with respect to HRQOL and coping.

**Methods:**

A total of 115 patients (91 women) with SHR were asked to answer five questionnaires: a study-specific questionnaire, the Chemical Sensitivity Scale for Sensory Hyperreactivity (CSS-SHR), the NHP, the SF-36, and the Jalowiec Coping Scale-60.

**Results:**

Eighty-three patients (72%; 70 women) completed all questionnaires. The SF-36 scores were less skewed and more homogeneously distributed and showed fewer floor and ceiling effects than the NHP scores. The SF-36 was also discriminated better between patients with high and low CSS-SHR scores. The reliability standard for both questionnaires was satisfactory. There were no gender differences in HRQOL. Patients with SHR had significantly lower HRQOL scores than the normative data in comparable domains of the NHP and the SF-36: emotional reactions/mental health, energy/vitality, physical mobility/functioning, and pain/bodily pain. In social isolation/functioning, the results were different; the NHP scores were similar to the normative data and the SF-36 scores were lower. The most commonly used coping styles were optimistic, self-reliant, and confrontational. Women used optimistic coping more than men. Compared with the normative group, patients with SHR used confrontational and optimistic coping more and emotive coping less.

**Conclusions:**

The current findings showed that both the NHP and the SF-36 were reliable instruments; but the results suggest that the SF-36 is a more sensitive instrument than the NHP for elucidating HRQOL in patients with SHR. Patients with SHR experienced a poor HRQOL and they followed the Western tradition of preferring problem-focused coping strategies to palliative and emotive strategies.

## Background

Sensory hyperreactivity (SHR) was found to be one explanation for airway symptoms induced by chemicals and scents. An objective test method called the capsaicin inhalation cough test and a questionnaire, the Chemical Sensitivity Scale for Sensory Hyperreactivity (CSS-SHR) have been developed to identify patients with SHR, and a high CSS-SHR score is directly related to capsaicin sensitivity [1,2]. The symptoms of SHR often mimic asthma and/or allergy, but in most cases asthma and allergy tests are negative, and asthma or allergy medication has no effect. The most common symptoms reported by the patients are cough, heavy breathing, difficulty getting air, chest weight, phlegm, hoarseness, stuffy nose, and eye irritation. Some patients also reported more general symptoms such as sweating, dizziness, and headache. Common trigger factors are perfume, flowers, coloured paints, cigarette smoke, and car exhaust fumes, as well as cold air and exercise [3]. More than 6% of the adult population in Sweden has been estimated to have SHR, where the SHR diagnosis was based on a high CSS-SHR score in combination with a positive reaction to the capsaicin inhalation cough test [2].

Health-related quality of life (HRQOL) is a measure of how diseases and symptoms affect health, well-being, and the ability to function in daily life. It includes several dimensions: physical function, role function, e.g., work, home management, social function, psychological function, and general well-being [4]. HRQOL is measured by means of standardized questionnaires, which can be generic or disease-specific. Generic instruments cover a wide range of dimensions and are applicable in a wide variety of conditions. Disease-specific instruments, on the other hand, are designed for a particular patient population. The choice of instrument depends on the approach of the study, and it is important to use instruments that are both reproducible and valid [5]. Generic HRQOL instruments need to satisfy different criteria to be suitable for measuring health outcomes in clinical settings and research. One important criterion is that the instrument should be validated (assessed to ensure it measures what it purports to measure). One aspect of validity refers to construct validity (the ability of an instrument to distinguish between known groups). Another important characteristic is reliability (the degree to which an instrument is free from random error and all items measure the same underlying attribute). Further the instrument must be acceptable to patients, and quick and easy to use [6]. The Nottingham Health Profile (NHP) and the Short-Form 36 Health Survey (SF-36) are both generic instruments that address multidimensional aspects of HRQOL [7].

The NHP questionnaire has been used in several studies of chronic illnesses and conditions [8,9]. The questionnaire was developed in 1980 at Nottingham University (UK) for measuring subjective health status [10]. Some limitations have been shown with regard to sensitivity. Patients with milder symptoms tend to score zero (no problems) or near to zero, and the questionnaire seems, therefore, to be unsuitable for use in examining improvements [11]. The SF-36 questionnaire was developed 10 years later from a questionnaire known as the Medical Outcome Study General Health Survey Instrument [12]. It has also been used in several studies of various chronic illnesses and conditions [8,9,13]. A limitation has been found in the bodily pain dimension in SF-36; it only correlates moderately to a pain scale that quantifies pain severity in patients with hip and knee osteoarthritis [14].

Two previous studies, which used the NHP, have described the HRQOL in patients with SHR [3,15]. The condition has great influence on several aspects of daily life and the reduced HRQOL did not change during a five-year period [3]. Millqvist el al. showed that patients with SHR with reduced HRQOL were more sensitive to inhaled capsaicin [15]. Using a qualitative approach, Larsson et al. studied a group of patients with SHR and how they handle their problems. The patients felt that there was a lack of understanding from others, felt limitations in daily life and were afraid that the symptoms would indicate a problem that would become a serious disease [16]. Söderholm et al. reported similar results in patients with SHR in regard to their limitation in participating in society and lack of understanding from others [17]. Similar results have been found in patients with chemical sensitivity [also called multiple chemical sensitivity (MCS)]; the results showed a lack of access regarding education, use of public transportation, visits to restaurants, movies, friends, medical care, and problems at work [18-20]. Further, according to Lipson, people have difficulties living with MCS. They have to face social, attitudinal, and logistical barriers. Social suffering increased for people with MCS because their relationships with family, friends, school or work, and physicians were negatively affected [21].

When people become patients, they enter a stressful situation. Coping is the response that aims to reduce the level of stress. Coping is an attempt to manage situations that produce discomfort, and it is a function of both the environment and the individual’s cognitive appraisal of the situation. According to this theory, the individual and the situation cannot be separated: coping is not outcome dependent; rather it is simply the attempt to manage the situation [22]. The Jalowiec Coping Scale-60 (JCS-60) was developed in 1987 to measure the process of coping [23]. It has been used in several studies in different conditions [24,25], and studies show that women may use different kinds of coping strategies than men [26,27]. Depending on their function, coping strategies can be classified in two major categories. One is problem-focused, in which the patient deals directly with the problem, e.g. information, seeking, and goal setting. The other is emotion-focused coping, in which the patient deals with the emotions associated with the stress, e.g., worry or depression. According to Lazarus, in Western culture there is a strong tendency to regard problem-focused coping as being more successful and effective than emotive coping [22].

Nordin et al. studied coping strategies in a group of patients who considered themselves intolerant to odorous/pungent chemicals. The most commonly used and effective coping strategies were avoiding odorous environments, and asking people to limit their use of odorous substances. Other strategies were to accept the situation and reprioritize how important things were [28]. Patients with asthma-like symptoms describe having similar symptoms and trigger factors as SHR-patients. Ringsberg et al. studied the lives of a group of female patients with asthma-like symptoms. The patients had few coping strategies; they experienced social isolation and distress and a feeling of ‘walking around in circles’ [29]. A study with problem-based learning in patients with asthma-like symptoms showed that the participants learned to use new coping strategies, could describe the disorder in words, and had their self-confidence increase [30]. Larsson et al. showed that patients with SHR cope with their symptoms by avoiding situations that they cannot tolerate. Another way they managed difficult situations was by disparaging the symptoms or to simply denying them [16].

Little is known about HRQOL and which of the two generic instruments, the NHP or the SF-36, is more suitable for measuring HRQOL in patients with SHR. Further, little is known about coping strategies among patients with SHR. In addition, little is known about gender differences and differences between normative data in HRQOL and coping among these patients. These issues can, however, be examined using established questionnaires.

The aims of the present study of patients with SHR were (1) to compare two generic quality-of-life questionnaires, the NHP and the SF-36, regarding their suitability (in respect of floor effects, ceiling effects, validity and reliability), and acceptability (assessed by using the completion rate as an indicator) as outcome measures of HRQOL; (2) to evaluate how these patients cope with the illness; (3) to assess whether there are differences between women and men with respect to HRQOL and coping, and (4) to assess whether there are differences between patients and normative data with respect to HRQOL and coping.

## Methods

### Study design

This study was designed as a comparative study and data were collected using questionnaires administered to patients who had visited the clinic and been diagnosed with SHR. All patients were selected from the medical records at the Department of Allergology at the Sahlgrenska University Hospital in Gothenburg, Sweden, between January 2007 and October 2009.

### Participants

One-hundred and fifteen patients (91 women and 24 men) fulfilled the criteria for SHR and were invited to participate in this study. Inclusion criteria were: (1) a history of airway symptoms induced by chemicals and scents, (2) a positive reaction to the capsaicin inhalation test, as described by Johansson et al. [1], (3) no sign of bronchial reversibility or variability according to spirometry, (4) a negative reaction to the methacholine inhalation test, administered according to international guidelines [31], and (5) a negative skin prick test, with a standard panel of 10 allergen sources common to Sweden. Exclusion criteria were allergy, rhinitis, post-nasal drip, and gastroesophageal reflux disease (GERD), as were use of angiontensin-converting enzyme inhibitor or medication for GERD and current smoking. Informed consent was obtained from all participants at the start of the investigation. The study was approved by the Regional Ethical Review Board of Gothenburg, Sweden.

### Data collection

Data were collected using a study-specific questionnaire and four standardized questionnaires [the CSS-SHR, NHP, SF-36, and JCS-60]. The questionnaires were sent by mail to the patients with a covering letter, an informed consent form, and a prepaid envelope. The estimated time to complete all five questionnaires was about 25 minutes. The patients were reminded about the study once, within a month, and a new letter and questionnaires were sent to them. In some cases, patients were phoned for supplementary answers. The patients were asked to answer the questions based on their condition as experienced during the previous month. Permission to use the instruments was obtained from the respective copyright holders. All data were recorded by subject number so the authors and the statistician did not know the identity of the patients.

### Questionnaires

#### Study-specific questionnaire

The study-specific questionnaire contains questions regarding demographic data (age, gender, duration of symptoms, smoking habits, and inducing factors).

#### Standardized questionnaires

##### CSS-SHR

The CSS-SHR questionnaire was developed from the chemical sensitivity scale in 2004 to quantify self-reported sensitivity to chemicals and scents in the course of daily activities. It is validated and has good reproducibility [32]. The questions were selected from a large number of items about odour intolerance and consist of 11 statements/questions that are particularly sensitive for differentiating patients with SHR from healthy controls [33]. The unweighted sum of all 11 items makes up the individual’s total CSS-SHR score, ranging from 0 to 54. A score of ≥ 43 points is regarded as a diagnostic cut-off value for SHR, and a high score indicates high sensitivity to chemicals and scents [32]. In the adult Swedish population the prevalence of such odour intolerance, defined as a CSS-SHR score ≥ 43, has been determined to be 19%, with an increased risk for female gender (odds ratio: 2.3) [34].

##### NHP

The NHP is a two-part instrument. In order to compare the NHP and the SF-36, only part one of the NHP was used in this study. Part I contains 38 items covering six aspects of HRQOL, concerning the domains of emotional reactions, energy, physical mobility, social isolation, pain, and sleep. The response alternatives for each item are ‘yes’ and ‘no’ , depending on whether that item fits the individual’s current situation. The possible score for each dimension ranges from zero (no problems at all) to 100 (presence of all problems within the area) [10]. The NHP has shown to have good reliability and validity [35,36].

##### SF-36

The SF-36 contains 36 items covering eight health concepts: mental health, vitality, psychical functioning, social function, bodily pain, role limitations due to physical problems, emotional problems, and general health. The response options used were 2-graded (1 = yes, 2 = no), and 3-graded (1 = yes, much limited, 3 = no, not at all limited), 5-graded (1 = not at all, 5 = very much), and 6-grade (1 = all the time, 6 = none of the time) scales. The scores for each scale are coded, summed, and transformed into a scale ranging from 0 (worst possible health) to 100 (best possible health) [12,37]. In studies, the SF-36 has shown good reliability and validity [38,39].

##### JCS-60

The JCS-60 was used to assess general coping behaviour [23]. It consists of two parts (A = use, B = effectiveness) with 60 items in each part. Each item describes a strategy a person can use to handle stress. Strong positive correlations between part A (use) and part B (effectiveness) for JCS-60 were demonstrated in patients with end-stage renal disease, and in people with kidney transplants, which may indicate that these two parts reflects similar or the same aspects of coping [40,41]. Therefore only part A was used in this study. The participants were asked to think of something stressful in connection with their sensitivity to chemicals and scents and to rate how often they used the strategy, on a 4-point scale (0= never used, 3 = often used). The coping strategies are grouped into eight styles: optimistic, self-reliant, confrontational, fatalistic, evasive, supportive, palliative, and emotive. The JCS-60 has been comprehensively evaluated and found to have good reliability and validity [23,42].

### Statistical analysis

Descriptive statistics were used to examine the data. Data are presented for continuous variables as mean and standard deviation (SD) and for categorical variables as percent (%).

For comparison between groups, Fisher’s Exact Test was used for dichotomous variables, and the Mann–Whitney U-test and unpaired t-test were used for continuous variables. Results were considered significant if p < 0.05. Analysis of comparability between the NHP and the SF-36 were calculated between the domains that are comparable, including emotional reactions, energy, physical mobility, social isolation, and pain for the NHP, and mental health, vitality, physical functioning, social functioning, and bodily pain for the SF-36 (Table 1). The level of correlation between the NHP and the SF-36 was calculated among the domains’ scores with the Pearson Correlation Coefficient. To compare the mean scores per dimension between the NHP and the SF-36 questionnaire, the NHP score was subtracted from 100, since the scoring pattern in this instrument works in the reverse fashion to that of the SF-36. Hence, the score in the present study for both the NHP and SF-36 are presented in a range from 0 to 100 (0 = worst perceived health and 100 = best possible health).

**Table 1:** Different domains in the NHP and the SF-36

**Domains**	**NHP**	**SF-36**
Psychological status	**Emotional reactions (9 items)**	**Mental health (5 items)**
	**Energy (3 items)**	**Vitality (4 items)**
Physical activity	**Physical mobility (8 items)**	**Physical functioning (10 items)**
Social activity	**Social isolation (5 items)**	**Social functioning (2 items)**
Pain	**Pain (8 items)**	**Bodily pain (2 items)**
Others	Sleep	Role-physical
		Role-emotional
		General health

Comparable domains are shown in bold.

In order to determine suitability of the NHP and SF-36 questionnaire to measure HRQOL, the percentages of patients obtaining the worst possible score of 0 (floor effect) and the best possible scores of 100 (ceiling effect) were calculated. Further, validity of the NHP, SF-36, and JCS-60 questionnaires were examined by the Mann–Whitney U-test regarding the relative ability to discriminate among patients with high (≥ 43 points) and low (< 43 points) CSS-SHR scores. Reliability was expressed as Cronbach’s coefficient alpha (α). A coefficient > 0.70 was taken to be acceptable and satisfactory reliability [43].

Norm and reference values distributed for age and sex based on data from larger population studies are used for the NHP, the SF-36 and the JCS-60 questionnaires [26,37,44].

All statistical analyses were performed using software, StatView 5 (SAS Institute INC., Cary, NC, USA) and SPSS 17.0 (SPSS, Inc., Chicago, IL, USA).

## Results

### Participant characteristics

Eighty-three (72%) patients returned the questionnaires. Reasons for not participating were language difficulties in 5 patients (2 men) and personal reasons in 27 patients (9 men). The group mainly consisted of women. Thirteen men (15.6%) participated in this study. The demographic data of the study group are shown in Table 2. There were no significant gender differences with respect to age, lung function, body mass index, symptom duration, and smoking habits. Women reported exercise as a trigger factor significantly more than men (p < 0.05); otherwise, there were no gender differences in reported trigger factors.

**Table 2:** Characteristics of the study group

**Characteristics**	**Subjects (*n *=83)**
Sex, Female/Male (*n*)	70/13
Age (years)	53.1 (11.8)
Duration of symptoms (years)	13.6 (10.2)
FEV_1_ % predicted	99.8 (12.9)
BMI (kg/m^2^)	25.2 (3.8)
CSS-SHR	43.7 (7.9)
**Smoking status (%)**	
Never	69.0
Previous	31.0
**Trigger factors (%)**	
Chemicals and scents	100
Cold air	71.4
Exercise	70.2

Data are shown as mean and standard deviation (SD) for age, duration of symptoms, FEV_1_, BMI, CSS-SHR, and otherwise as percentage (%).

Definition of abbreviations: FEV_1_ = Forced expiratory volume in one second; BMI = Body mass index; CSS-SHR = the Chemical sensitivity scale for sensory hyperreactivity.

### Questionnaires

#### CSS-SHR

The mean (SD) CSS-SHR score for the whole group was 43.7 (7.9). There were no differences in the scores between men and women. The patients were divided into two groups according to the cut-off value for CSS-SHR: those who had a score of ≥ 43 points (high score) and those with lower scores (low score). Forty-seven patients (57%; 6 men) had a score of ≥ 43 points. There were no differences in the NHP and the JSC-60 between patients with high and low scores for the CSS-SHR. For the SF-36, patients with high CSS-SHR-scores reported significantly more problems with role limitations due to psychical problems (p < 0.05) and general health (p < 0.02) than the low-scoring group.

#### NHP and SF-36

**Figure 1:**
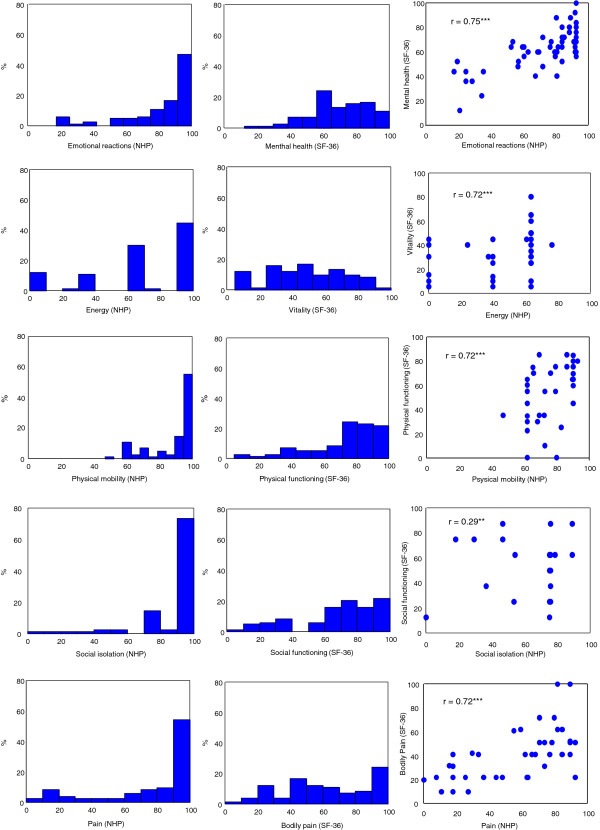
**Frequency distribution of scores on comparable domains of the NHP (left panel) and the SF-36 (middle panel). Right panel shows correlation of comparable domains of the NHP and the SF-36.** *** p < 0.001; ** p < 0.01.

The HRQOL did not differ between men and women. Comparison of the frequency distribution of the NHP and the SF-36 showed that the NHP scores were more skewed than the SF-36 scores (Figure 1). The prevalence of patients for the worst possible scores (floor effects) was higher for the NHP scale (range 0–12.0%) compared with the SF-36 (range 0–1.2%). There were no differences between the sexes in floor or ceiling effects. The prevalence of patients with best possible scores (ceiling effects) was also higher for the NHP scale (range 28.9-73.5%) than for the SF-36 score (range 1.2-24.1%). All domains in the two instruments were statistically reliable, with α values of > 0.70, except for energy as assessed by the NHP (α = 0.69). The SF-36 showed the highest reliability of the two instruments, attaining α values of 0.95 for bodily pain and 0.91 for physical functioning (Table 3).

**Table 3:** **Floor and ceiling effect and Cronbach’s α values in comparable domains of the NHP and the SF-36 scales (*n *=83)**

**NHP**	**Floor**	**Ceiling**	α values*	**SF-36**	**Floor**	**Ceiling**	α values*
Emotional reactions	0	28.9	0.76	Mental health	0	2.4	0.80
Energy	12.0	44.6	0.69	Vitality	0	1.2	0.88
Physical mobility	0	55.4	0.71	Physical functioning	0	6.0	0.91
Social isolation	1.2	73.5	0.71	Social functioning	1.2	21.7	0.86
Pain	0	51.8	0.89	Bodily pain	1.2	24.1	0.95

Data are presented as percentages *Cronbach’s α values.

The levels of association between the scores for corresponding dimensions on both questionnaires are shown in Figure 1. High correlations (r > 0.70) were found between comparable subscales of the two instruments in all areas except for social isolation/social functioning (r = 0.29).

As Table 4 shows, the patients reported significantly lower HRQOL than the reference group in the NHP questionnaire for emotional reactions, energy, physical mobility and pain. There were no differences between patients and the reference group for social isolation and sleep. In the SF-36, patients reported significantly lower HRQOL than the reference group, in all dimensions of the questionnaire.

**Table 4:** Comparisons of NHP, SF-36 and JCS-60 mean scores between patients with SHR and reference values

**Instruments and scales**	**Study group**	**Reference values**	**P-values**
	**Mean scores (SD)**	**Mean scores (SD)**	
**NHP (*n *= 83)**			
Emotional reactions	81.5 (22.0)	89.0 (2.5)	0.002
Energy	69.0 (33.9)	83.0 (6.6)	<0.001
Physical mobility	89.2 (14.7)	93.2 (5.3)	0.02
Social isolation	90.0 (20.6)	94.3 (1.9)	0.06
Pain	78.3 (30.2)	91.0 (5.3)	<0.001
Sleep	75.3 (30.4)	81.9 (6.5)	0.06
**SF-36 (*n *= 83)**			
Mental health	69.2 (18.0)	80.0 (1.2)	<0.001
Vitality	48.6 (24.5)	67.4 (2.4)	<0.001
Physical functioning	74.2 (22.6)	82.4 (7.7)	0.002
Social functioning	68.9 (26.7)	87.3 (1.7)	<0.001
Bodily pain	60.3 (29.4)	70.0 (4.1)	0.004
Role-physical	58.7 (41.2)	78.2 (8.5)	<0.001
Role-emotional	69.9 (38.5)	83.6 (4.4)	0.002
General health	50.4 (23.7)	71.9 (5.3)	<0.001
**JCS-60 (*n *= 81)**			
Optimistic	1.84 (0.6)	1.59 (0.1)	<0.001
Self-reliant	1.58 (0.6)	1.60 (0.1)	0.72
Confrontational	1.83 (0.6)	1.60 (0.1)	0.001
Fatalistic	1.13 (0.6)	1.26 (0.1)	0.06
Evasive	1.19 (0.5)	1.11 (0.1)	0.19
Supportive	1.19 (0.6)	1.27 (0.2)	0.26
Palliative	1.03 (0.5)	0.97 (0.1)	0.28
Emotive	0.82 (0.5)	1.02 (0.2)	0.001

#### JCS-60

Two patients did not answer the JCS-60 because of difficulty understanding the questions. The patients reported most frequently using optimistic, self-reliant, and confrontational coping methods. The methods they used least were palliative and emotive coping. Women used significantly more optimistic coping than men (p < 0.05); otherwise there were no gender differences. All subscales were statistical reliable with α values of > 0.70, except for fatalistic, supportive, palliative, and emotive coping styles. Patients’ coping styles are shown in Table 5.

**Table 5:** **The Jalowiec Coping Scale-60 (JCS-60) for women and men (*n *=81)**

**Scales**	**Total (n=81)**	**Women (n=68)**	**Men (n=13)**	**P-values**	**Chronbach’s α**
	**Mean (SD)**	**Mean (SD**	**Mean (SD**		
Optimistic	1.84 (0.6)	1.90 (0.5)	1.51 (0.6)	0.03	0.76
Self-reliant	1.58 (0.6)	1.61 (0.7)	1.42 (0.5)	0.19	0.75
Confrontational	1.83 (0.6)	1.83 (0.6)	1.82 (0.4)	0.61	0.82
Fatalistic	1.13 (0.6)	1.17 (0.6)	0.90 (0.7)	0.16	0.55
Evasive	1.19 (0.5)	1.20 (0.5)	1.12 (0.4)	0.63	0.72
Supportive	1.19 (0.6)	1.16 (0.6)	1.32 (0.6)	0.40	0.59
Palliative	1.03 (0.5)	1.07 (0.5)	0.81 (0.5)	0.06	0.58
Emotive	0.82 (0.5)	0.85 (0.5)	0.66 (0.5)	0.33	0.62
Total score	1.33 (0.4)	1.35 (0.4)	1.20 (0.4)	0.11	0.91

When matched for norm values, the patients used significantly more optimistic and confrontational coping and less emotive coping. There were no differences between patients and the reference group in the other coping styles (self-reliant, fatalistic, evasive, supportive, and palliative) (Table 4).

## Discussion

The main results in the present study can be summarized as follows. First, the results indicate that the SF-36 scores were less skewed and more homogeneously distributed than the NHP scores. The SF-36 showed less floor and ceiling effects compared with the NHP. The SF-36 was also better for discriminating between patients with high and low CSS-SHR scores with regard to role limitations due to psychical problems and general health. The reliability standard for both questionnaires was satisfactory. No gender differences in HRQOL were measured with the two instruments. Second, the most commonly used coping styles were optimistic, self-reliant, and confrontational coping. Women used optimistic coping significantly more than men. Third, patients with SHR had significantly higher impairment in several dimension of HRQOL compared with the reference group. Further, the patients used optimistic and confrontational coping more and emotive coping less, compared with the reference group.

Both the NHP and the SF-36 have been compared in several studies in patients with different diseases [8,9]. The NHP has been used in patients with SHR [3,15], but the SF-36 has never been used in this group of patients. To assess the utility of these instruments as a general outcome measure of HRQOL, different criteria have to be taken into consideration. It has to be quick and easy to use. Both HRQOL questionnaires fulfilled these criteria, taking about 10 min to complete. The instrument should also be acceptable to respondents with few missing values, which was indicated with the high response rate of 72% in the present study, and showing only one or few missing values among the participants in both questionnaires. Further, the availability and the cost of using a questionnaire are also important factors. Some advantages with the NHP are that it is readily available and inexpensive to use. The SF-36, on the other hand, is strictly copyrighted, and is rather expensive to administer because of a user fee [45].

The patients NHP and SF-36 scores differed to the same extent from the reference values, with significantly lower scores (more difficulties) in comparable domains: emotional reactions/mental health, energy/vitality, physical mobility/functioning, and pain/bodily pain. This is in line with a study in patients with SHR and in a study of long-term survivors after a myocardial infarction [15,46]. In social isolation/functioning, the results were different, with NHP scores similar to normative data and SF-36 scores significantly lower. The result in social isolation is in contrast to a longitudinal study in patients with SHR, showing a greater impairment in social isolation, compared with reference values [3]. There were no differences from reference values in sleep, the other NHP score. All other SF-36 scores were also lower and differed significantly from reference values, social functioning, role-physical, role-emotional, and general health.

The present NHP results showed a higher prevalence of ceiling effect (indicating best possible quality of life) in all dimensions compared with the SF-36 results, but also a higher prevalence of floor effects in energy (indicating lowest possible quality of life). Otherwise, there were minor floor effects in both the NHP and the SF-36. This is in accordance with other studies in patients with chronic limb ischemia [8,47] and in patients with chronic obstructive disease [48], which showed fewer ceiling and floor effects in the SF-36 compared with the NHP. The advantages with the SF-36 may depend on each item having different possible scores, whereas the NHP items are dichotomous with only a yes/no alternative, providing more possibilities for results at the extreme ends of both good and ill health. To use a score with only a yes/no alternative may also make it difficult to show improvement over time. The findings of Chronbach’s coefficient α values of ≥ 0.70 in all but one dimensions of HRQOL implies good internal consistency for both questionnaires in accordance with earlier findings [8,47], but the SF-36 seems more preferable because it has the highest α values.

As mentioned, the CSS-SHR questionnaire can be used to quantify self-reported sensitivity to chemicals and scents in the course of daily activities [32]. Our results showed that patients with high CSS-SHR score reported significantly more problems with role limitations due to psychical problems and general health than those with low CSS-SHR score, measured with the SF-36. This is in line with Brown et al., showing the SF-36 to be more sensitive than the NHP in detecting the impact of breathlessness in patients after a myocardial infarction [46]. In contrast, Wann-Hansson et al. demonstrated that patients with critical leg ischemia had more problems with pain and physical mobility before revascularization than those with intermittent claudication, measured with the NHP [8]. Similar results have been found in patients with chronic limb ischemia, showing NHP to be more sensitive in detecting problems with pain and psychical mobility [47]. Nevertheless, Prieto et al. found that both instruments are similar in discriminating among different levels of respiratory impairment [48]. However, the SF-36 seems to have more validity in discriminating among levels of chemical sensitivity in patients with SHR. The SF-36 results are less skewed and more homogeneously distributed, which may suggest that it is more sensitive to explain HRQOL in patients with SHR, with respect to psychical problems and general health.

The patients in the present study used optimistic and confrontational coping significantly more and emotive coping significantly less often than the reference values. This is in line with the results of Lindqvist et al., who found that people with kidney transplants used optimistic coping significantly more and emotive coping significantly less often than the general population [41]. Further, the most frequently used coping styles among the patients were optimistic, self-reliant and confrontational. The two least used coping styles were palliative and emotive. This is in line with results in patients on continuous ambulatory peritoneal dialysis [40], people with kidney transplants [41], and patients with myocardial infarction (MI) [13]. Kristofferzon et al. found that over a 12-month period the most used coping methods after an MI were optimistic, self-reliant and confrontational and the least used methods were palliative and emotive [25]. The Chronbach’s coefficient α values of ≥ 0.70 was only found in four out of eight coping styles (optimistic, self-reliant, confrontational and evasive). The results are in accordance with a Swedish population study [26] and in patients after an MI [13].

### Limitations

Some limitations of this study were the small sample size and the selection of participants from only one allergy specialist clinic. Further, the study consisted of mainly female patients, which may limit the generalizability of the results. All patients who fulfilled the inclusion criteria’s were selected during a specific period. Hence, the study comprised only 15.6% male patients. This is, however, in accordance with earlier studies in patients with SHR, which showed a predominance of women [2,34]. Another limitation was having participants complete the questionnaires at home because this meant we did not know whether the questionnaires were answered without any external influences. To reduce the risk of occurring and to confirm the present results, a study is needed that includes a larger group of patients with SHR from where the patients are seen in different clinical settings, and having them complete the questionnaires in a clinical setting. In addition, longitudinal studies are required to examine HRQOL and coping in patients with SHR.

To be able to test-retest its reproducibility, an instrument has to be completed twice. Another limitation of the present study was that the questionnaires were only answered once, and therefore we were not able to assess the reproducibility of the questionnaires. However, in most respects, studies for group-level application have shown good reproducibility for the CSS-SHR [32], NHP and SF-36 [9,36,47]. On the other hand, no study concerning test-retest reproducibility of the JCS-60 has been found in the literature, and a future challenge would be to conduct such studies in healthy control subjects and in patients with different diseases.

In this study we only used generic questionnaires to measure HRQOL. One general recommendation is to use a generic quality of life questionnaire to compare results from different diseases and conditions. Disease-specific scales are required to discriminate between levels of severity of conditions or diseases and to detect important clinical changes. Therefore, a recommendation is often made to use both a generic questionnaire and a disease-specific questionnaire to obtain a HRQOL outcome [45]. However, no currently accepted disease-specific questionnaire exists for patients with SHR. Further research has to be conducted to develop an instrument to measure HRQOL in patients with SHR.

A further limitation of the study may be that we only used part A of the JCS-60 questionnaire. However, this is in accordance with studies in patients with MI and chronic illness [13,25,49]. As mentioned before, studies have shown a strong correlation between part A and B, which may suggest a risk that the use and efficiency components measure the same aspect of coping [40,41].

## Conclusions

The current findings showed that both the NHP and the SF-36 were reliable instruments; but the results suggest that the SF-36 is a more sensitive method than the NHP to elucidate HRQOL in patients with SHR. Patients with SHR experienced a lower HRQOL, and used more optimistic and confrontational coping, compared with normative data. They followed the Western tradition of preferring problem-focused coping strategies to palliative and emotive strategies.

## Abbreviations

α: Cronbach’s coefficient alpha; BMI: Body mass index; CSS-SHR: Chemical sensitivity scale for sensory hyperreactivity; GERD: Gastroesophageal reflux disease; FEV1: Forced expiratory volume in one second; HRQOL: Health-related quality of life; JCS-60: Jalowiec coping scale-60; MCS: Multiple chemical sensitivity; NHP: Nottingham health profile; SD: Standard deviation; SF-36: Short-form 36 health survey; SHR: Sensory hyperreactivity.

## Competing interests

The authors declare that they have no competing interests.

## Authors’ contributions

Both authors made substantive intellectual contributions to the present study, and will take public responsibility of its content. ETH: conception and design; coordinated and analysed the data and drafted the manuscript. M-LK: conception and design; analysed the data and drafted the manuscript. Both authors have given final approval of the submitted version.
